# Acupuncture for asthma: Protocol for a systematic review

**DOI:** 10.1097/MD.0000000000007296

**Published:** 2017-06-30

**Authors:** Meng Li, Xing Zhang, Haipeng Bao, Chunlei Li, Peitong Zhang

**Affiliations:** a Department of Oncology, Guang’anmen Hospital, China Academy of Chinese Medical Sciences; b School of Graduates, Beijing University of Chinese Medicine, Beijing, China.

**Keywords:** acupuncture, asthma, protocol, systematic review

## Abstract

**Background:**

Asthma is a common chronic disease in both adults and children. Inhaled corticosteroids (ICS) and β_2_-agonists are the major medications treating asthma with many side effects. Acupuncture has been used in many diseases including asthma. We aim to assess the efficacy and safety of acupuncture for asthma.

**Methods:**

The following electronical databases will be searched from inception to January 1, 2017: Medline, EMBASE, Cochrane Central Register of Controlled Trials (CENTRAL), SinoMed, the China National Knowledge Infrastructure Database (CNKI), the Chinese Scientific Journal Database (VIP database), and the Wanfang database. We will also search reference lists of identified studies, potential gray literatures, relevant conference abstracts, and registers of clinical trials. Two reviewers will independently undertake study selection, data extraction, and quality assessment. Data will be synthesized by either the fixed-effects or random-effects model according to a heterogeneity test. Changes in lung function will be assessed as the primary outcome. The level of control, medication usage, quality of life, exacerbations, symptoms, adverse events will be evaluated as the secondary outcomes. The RevMan V.5.3.5 will be employed for meta-analysis. Continuous outcomes will be presented as mean difference or standard mean difference, while dichotomous data will be expressed as relative risk.

**Results:**

This study will provide a high-quality synthesis of current evidence of acupuncture for asthma from several aspects including lung function, the level of control, medication usage, quality of life, exacerbations, symptoms and adverse events.

**Conclusion:**

The conclusion of our study will provide updated evidence to judge whether acupuncture is an effective intervention for children with asthma.

**Ethics and dissemination:**

It is not necessary for ethical approval because individuals cannot be identified. The protocol will be disseminated in a peer-reviewed journal or presented at a relevant conference.

**PROSPERO registration number:**

PROSPERO CRD42017054562.

## Abstract 2

Supplemental Digital Content is available in the text

## 1 Introduction

Asthma is a heterogeneous disease, usually characterized by chronic airway inflammation. It is defined by the history of respiratory symptoms such as wheeze, shortness of breath, chest tightness, and cough that vary over time and in intensity, together with variable expiratory airflow limitation.^[1]^ Both symptoms and airflow limitation are often triggered by factors such as viral respiration infections, exercise, allergen, or change in weather.^[2]^ There are estimated 300 million individuals affected by asthma around the world.^[3–5]^ And asthma is the most common chronic disease of childhood.^[6]^ Prevalence of current asthma in children aged 13 to 14 years ranges from 1.5% (Nepal) to 15.6% (Isle of Man).^[7]^ It is estimated that 346,000 people died of asthma worldwide every year.^[8]^

Achieving good control of symptoms is one of the most important goals of asthma management.^[1]^ Poor control of asthma symptom substantially increases the risk of exacerbations.^[9]^ Exacerbations are the major cause of morbidity and mortality in asthmatic patients^[10]^ and lead to significant costs for health care systems^[11]^. In addition, exacerbations also seriously diminish the quality of life of patients and their family, especially in children such as limitations on daily activities, school absences, poor sleep quality.^[12–15]^ Inhaled corticosteroids (ICS) and β_2_-agonists are the major medications treating asthma, and symptoms can be controlled in most patients using them. However, both systemic and local side effects of them have been reported, such as calcium and phosphate metabolism with subsequent risk of osteoporosis, adrenocortical suppression, bruising and skin thinning, dysphonia, oropharyngeal candidiasis, thirst, increased heart rate, palpitations, hypokalemia, and so forth.^[16–19]^ This fact has led to increasing interest in nondrug strategies that can assist in improving symptom control and/or reducing future risk.

Acupuncture is a form of therapy which stimulates acupoints by inserting filiform needles. The selection of acupoints is based on the syndrome differentiation from the diagnosis according to traditional Chinese medicine (TCM) theory. Thus, the choice and number of acupoints are different from 1 patient to another. Although many types of acupuncture therapy have been used in clinical practice, the most common are manual acupuncture (MA) and electroacupuncture (EA). MA is a form of stimulation by lifting, thrusting, or rotating the needle manually to manipulate the de qi sensation (a sensation of soreness, heaviness, numbness, or distension), while EA stimulates acupoints by connecting the needles to a small electrical current.^[20]^

Acupuncture has been used to treat asthma in China and Western countries.^[21,22]^ Previous studies indicated that acupuncture could improve symptoms of asthma, lung function, and decrease medication dosages, and could be applied as an adjunct to conventional medications.^[23,24]^ In TCM theory, the mechanism of treating asthma by acupuncture is believed to regulate and balance the obstructive Qi which can result in health problem. In Western medicine, the mechanism still remains unclear. Studies involving animals and humans have indicated that acupuncture could modulate the immune system. An experiment reported that acupuncture can decrease the ovalbumin specific IgE level as well as the Th17 cytokine levels (IL-17A, IL-17F, and IL-22) in the serum of the experimental asthma mice.^[25]^ In a clinical study, patients treated by acupuncture were observed improvement of CD3+ cells and CD4+ cells, and decrease in IL-6 and IL-10.^[26]^ In addition, 1 study has demonstrated that acupuncture could reduce airway resistance in people with asthma.^[27]^

There are an increasing number of studies on acupuncture for respiratory disorders published with inconclusive results in recent years. Some trials reported acupuncture could improve the symptoms and decrease the use of ICS of asthma.^[28,29]^ A systematic review was published in 2004 with poor recommendations on acupuncture for patients with asthma.^[30]^ There have been at least 5 randomized controlled trials (RCTs) published from then on. We aim to systematically assess the efficacy and safety of acupuncture for asthma.

## 2 Methods

### 2.1 Inclusion criteria for study selection

#### 2.1.1 Types of studies

Only RCTs in English and Chinese will be included. Quasi-RCTs, and cluster-randomized trials will be excluded. And duplicate studies will be also excluded. Restriction of publication type will not be applied during study selection.

#### 2.1.2 Types of patients

Studies involving patients of any age with asthma will be included without limitations related to gender, race, and education status. Studies involving patients with gastroesophageal reflux, tracheomalacia, tuberculosis, chronic upper airway cough syndrome, inhaled foreign body, bronchiectasis, primary ciliary dyskinesia, congenital heart disease, bronchopulmonary dysplasia, cystic fibrosis, cardiac failure, medication-related cough, pulmonary embolism will be excluded.

#### 2.1.3 Types of interventions

##### 2.1.3.1 Experimental interventions

Acupuncture is defined as the stimulation of acupoints by needles, including manual acupuncture, electroacupuncture, ear acupuncture, scalp acupuncture, plum blossom needle, fire needling, or dermal needle. Studies using acupuncture in experimental groups will be included, regardless of the number, duration, and frequency of treatment sessions. Studies with other stimulation forms including moxibustion, laser acupuncture, transcutaneous electrical nerve stimulation, pharmacoacupuncture, or acupressure will be excluded.

##### 2.1.3.2 Control interventions

Studies of control groups using no treatment, placebo acupuncture (a special needle with blunt tip does not penetrate the skin, but a small pricking sensation is felt by the patient),^[20]^ sham acupuncture (acupuncture for an unrelated condition, superficial insertion at acupoint or non-acupoint locations), or an acupuncture-like intervention will be included. Studies comparing 2 different types of acupuncture or comparing acupuncture with Chinese herbal medicine will be excluded.

The following treatment comparisons will be investigated:

1. Acupuncture versus no treatment
2. Acupuncture versus placebo/sham acupuncture
3. Acupuncture versus other active therapies
4. Acupuncture with another active therapy versus the same therapy alone

#### 2.1.4 Types of outcome measures

##### 2.1.4.1 Primary outcomes

Changes in lung function (peak expiratory flow rates (PEFR), forced expiratory volume in one second (FEV1), forced vital capacity (FVC)) will be assessed as primary outcome.

##### 2.1.4.2 Secondary outcomes

The secondary outcomes of this review will include:

1. The level of control (e.g., the Childhood Asthma Control Test (C-ACT); the Asthma Control Test (ACT); GINA criteria; the Asthma Control Questionnaire (ACQ))
2. Medication usage
3. Quality of life (e.g., the Asthma Quality Life Questionnaire (AQLQ))
4. Exacerbations
5. Symptoms
6. Adverse events

### 2.2 Search methods for the identification of studies

#### 2.2.1 Electronic searches

We will search the following electronic databases from inception to January 1, 2017: Medline (via Ovid), EMBASE (via Ovid), Cochrane Central Register of Controlled Trials (CENTRAL), SinoMed, the China National Knowledge Infrastructure Database (CNKI), the Chinese Scientific Journal Database (VIP database), and the Wanfang database. The strategy was created based on a discussion among all reviewers according to the Cochrane handbook guidelines. The following search terms will be used: asthma, antiasthma, anti-asthma, respiratory sounds, wheeze, bronchial spasm, bronchospasm, bronchoconstriction, bronchial hyperreactivity, respiratory hypersensitivity, airway, lung, hypersensitivity, hyperreactive, allergy, insufficiency, dust, mite, acupuncture therapy, acupoints, acupuncture, manual acupuncture, electroacupuncture, electro-acupuncture, ear acupuncture, auricular acupuncture, scalp acupuncture, plum blossom needle, fire needling and dermal needle. The equivalent search words will be used in the Chinese databases. The search strategy for Medline (via Ovid) is shown in Table 1. And the other strategies for EMBASE, CENTRAL, SinoMed, CNKI, VIP and the Wangfang database are shown in Supplementary Tables 1–4

**Table 1:**
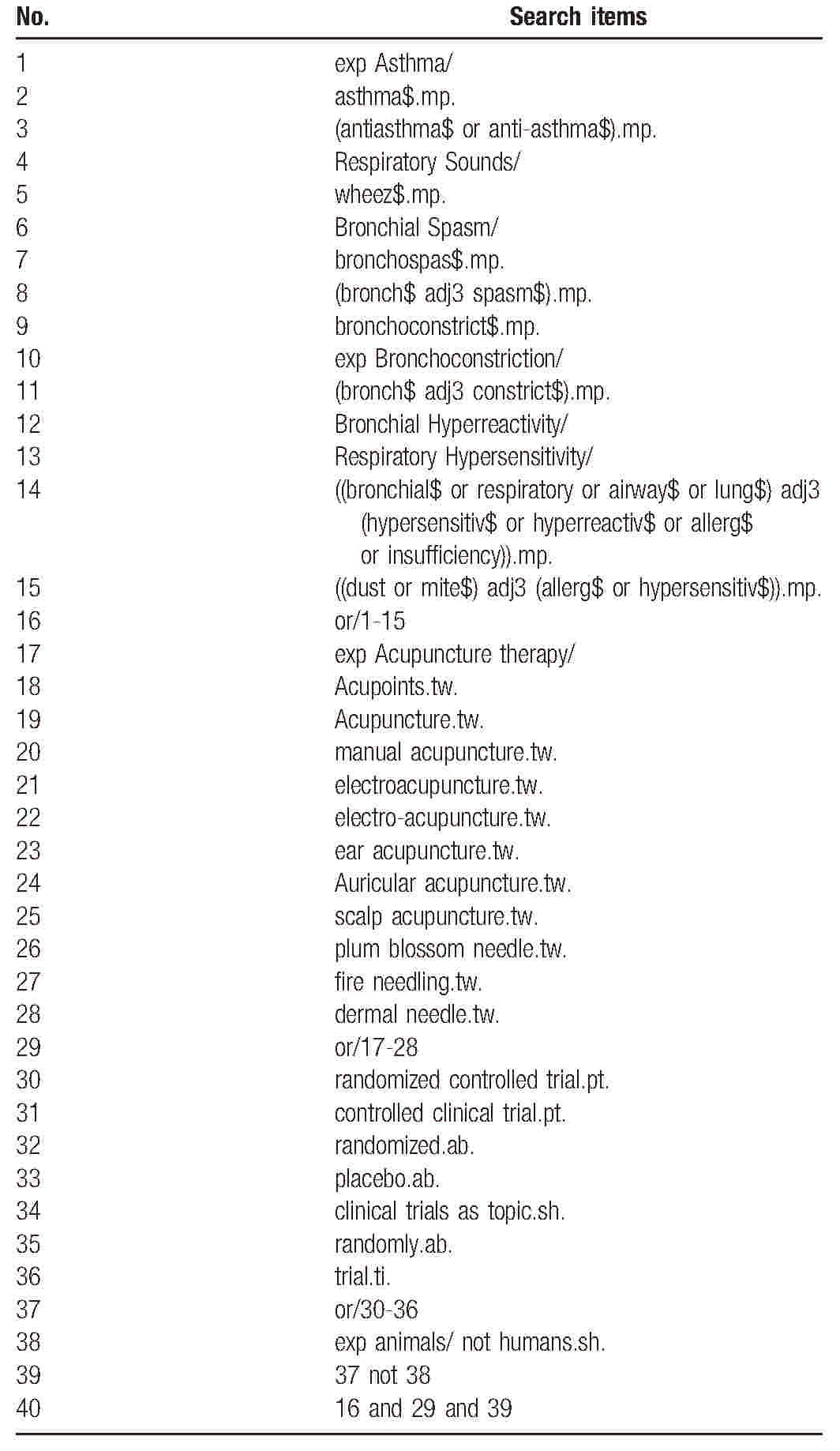
Search strategy used in Medline (via Ovid).

#### 2.2.2 Searching other resources

The reference lists of studies and systematic reviews related to asthma and acupuncture will be examined for additional trials. Potential gray literatures will be searched in OpenGrey.eu. We will search relevant conference abstracts for eligible trials. In addition, we will also search the ClinicalTrials.gov and the WHO International Clinical Trials Registry Platform (ICTRP) for all new reviews relevant to this topic.

### 2.3 Data collection and analysis

#### 2.3.1 Selection of studies

All review authors have received training to ensure a good understanding of the purpose and process of the review. Records from electronic databases and other resources will be uploaded to a database created by Endnote X7. Two review authors (HB and CL) will independently screen the titles, abstracts, and keywords of all retrieved records. Trials meeting inclusion criteria will be obtained for further assessment of full text. A table named “Reasons for excluded studies” will be used for recording excluded studies. We will resolve disagreements by consensus between the 2 review authors (HB and CL) or by involving a third review author (XZ).

#### 2.3.2 Data extraction and management

Two review authors (HB and CL) will independently extract data from the selected studies and fill in a data collection form that has been piloted on at least 1 study. Any disagreement will be solved by consensus or an arbiter (XZ). If needed, authors of trials will be contacted and be asked to provide incomplete data. We will extract the following data:

1. General information: the first author, time of publication, the source/journal.
2. Methods: study design, study duration, country, number of study centers, study setting, withdrawals/drop-outs.
3. Participants: sample size, mean age, age range, gender, severity of asthma, asthma diagnostic criteria, baseline lung function, inclusion criteria, and exclusion criteria.
4. Interventions: type of acupuncture/control, needles, number and name of acupoints, insertion depth, manipulation after insertion, “De-qi” required or not, duration of treatment, frequency of treatment, description of therapists’ qualifications.
5. Outcomes: primary and secondary outcomes specified and collected, and time points reported.
6. Notes: funding for trial, and notable conflicts of interest of trial authors.

#### 2.3.3 Assessment of risk of bias in included studies

The risk of bias in each study will be assessed by 2 independent authors (HB and CL) using the criteria outlined in the Cochrane Handbook for Systematic Reviews of Interventions. There are 7 domains in the Cochrane “Risk of bias tool”: random sequence generation, allocation concealment, blinding of participants and personnel, blinding of outcome assessment, incomplete outcome data, selective outcome reporting, and other bias. We will grade each potential trial of bias as low, unclear or high, and the judgement with a justification will be filled in the “Risk of bias” table. Any disagreement will be resolved by discussion or by involving an arbiter (XZ).

#### 2.3.4 Measures of treatment effect

For continuous data, we plan to present results as mean difference (MD) or standard MD (SMD) with 95% CIs. For dichotomous outcomes, data will be expressed as the relative risk (RR) with 95% CIs.

#### 2.3.5 Unit of analysis issue

Only data from the first experimental period in cross-over trials will be included to avoid carry-over effects. For studies with 1 or more intervention groups in common, we will combine all relevant experimental intervention groups of the study into a single group, and combine all relevant control intervention groups into a single control group to overcome a unit-of-analysis error.

#### 2.3.6 Dealing with missing data

In cases in which there are missing data, we will consider why the data are missing (missing at random or not). Whenever possible, we will try to contact the original investigators to request any inadequate and missing data of the included studies. If unable to obtain missing data, available case analysis will be performed (include data only on those whose results are known). And we will address the potential impact of missing data on the findings of the review in the Discussion section.

#### 2.3.7 Assessment of heterogeneity

We will assess heterogeneity by visually inspecting the forest plots to detect nonoverlapping CIs and by investigating *χ*^2^ (with *P* value >.10 indicating no heterogeneity) and *I*^2^ statistic. *I*^2^ ≥ 50% will be considered as representing substantial heterogeneity, while *I*^2^ < 50% will be taken as evidence of no heterogeneity. For substantial heterogeneity, we will explore possible causes by sensitivity analysis and subgroup analysis.

#### 2.3.8 Assessment of reporting biases

If the number of included studies is more than 10, we will generate funnel plots to detect reporting biases and small-study effects.

#### 2.3.9 Data synthesis

RevMan V.5.3.5 will be used for data analysis and synthesis when a meta-analysis is allowed. Continuous outcomes will be presented as MD/SMD with 95% CIs, while dichotomous data will be expressed as RR with 95% CIs. If *I*^2^ < 50%, the fixed-effects model will be used for data analysis. If *I*^2^ ≥ 50%, a random-effect model will be used. In addition, the sensitivity analysis and subgroup analysis will be employed for exploring the causes of heterogeneity. When meta-analysis is not applicable, narrative synthesis will be conducted.

#### 2.3.10 Subgroup analysis

We plan to carry out subgroup analyses if we identify substantial heterogeneity. The following subgroup analyses will be considered.

1. Age of patients (adults and children).
2. Different types of acupuncture therapies.
3. Degree of asthma severity.

#### 2.3.11 Sensitivity analysis

If data are sufficient, we will conduct sensitivity analyses for primary outcomes to test the robustness of the results. We will repeat the meta-analyses by excluding studies with high risk of bias of blinding and poor methodological quality to assess whether the quality of included studies influences the pooled results.

#### 2.3.12 Grading the quality of evidence

The quality of primary outcomes will be assessed by Grading of Recommendations Assessment, Development and Evaluation (GRADE).^[31]^ The assessments will be adjudicated into 4 levels: high, moderate, low, or very low.

## 3 Discussion

ICS and β_2_-agonists, associated with various side effects, are the major medications treating asthma. Global Initiative for Asthma (GINA) takes minimizing medication side-effects as one of the goals of asthma management. One study found that more than 60% of asthmatic children treated by ICSs experienced at least 1 side effect in daily life.^[32]^ Thus, nonpharmacological interventions are recommended to assist in improving symptom control and/or reducing future risk. With few side effects, acupuncture has been used for thousands of years in China. Although the mechanism in still unclear, the National Institutes of Health (NIH) suggested that acupuncture could be used as an adjunct treatment or an acceptable alternative or be included in a comprehensive management program in many diseases including asthma.^[33]^ We hope this review will provide more evidences of acupuncture for asthma.

There are some limitations in this review. Because the barrier of language, only studies published in English and Chinese will be included. Different types of acupuncture, age of patients and degree of asthma severity may run risk of heterogeneity. In addition, the measurements and tools of outcomes of included studies may be different.

The PRISMA-P checklist of the protocol is supplied in PRISMA_P_checklist.

## Supplementary Material

Supplemental Digital Content
