# Supplementary material for: Acupuncture for asthma: Protocol for a systematic review
Source: Medicine (Baltimore). 2017 Jun 30;96(26):e7296. doi: 10.1097/MD.0000000000007296 (PMC5500052; doi:10.1097/MD.0000000000007296)
Supplement: Supplemental Digital Content [file medi-96-e7296-s001.docx]

**Acupuncture for asthma: protocol for a systematic review**

**Meng Li, PhD^a,b^, Xing Zhang, PhD^b^, Haipeng Bao, PhD^b^, Chunlei Li, PhD^b^, Peitong Zhang, PhD^a,*^**

^a^Department of Oncology, Guang’anmen Hospital, China Academy of Chinese Medical Sciences, Beijing, China, ^b^School of Graduates, Beijing University of Chinese Medicine, Beijing, China.

*Correspondence should be addressed to:

Peitong Zhang: peitong_zhang@163.com

Supplementary Table 1. Search strategy used in Embase (via Ovid)

| **No.** | **Search items** |
| --- | --- |
| 1 | exp Asthma/ |
| 2 | asthma$.mp. |
| 3 | (antiasthma$ or anti-asthma$).mp. |
| 4 | Abnormal Respiratory Sound/ |
| 5 | Wheezing/ |
| 6 | wheez$.mp. |
| 7 | Bronchospasm/ |
| 8 | bronchospas$.mp. |
| 9 | (bronch$ adj3 spasm$).mp. |
| 10 | bronchoconstrict$.mp. |
| 11 | Bronchus Hyperreactivity/ |
| 12 | Respiratory Tract Allergy/ |
| 13 | ((bronchial$ or respiratory or airway$ or lung$) adj3 (hypersensitiv$ or hyperreactiv$ or allerg$ or insufficiency)).mp. |
| 14 | House Dust Allergy/ |
| 15 | ((dust or mite$) adj3 (allerg$ or hypersensitiv$)).mp. |
| 16 | or/1-15 |
| 17 | Exp Acupuncture/ |
| 18 | Acupuncture.tw. |
| 19 | Acupoints.tw. |
| 20 | manual acupuncture.tw. |
| 21 | electroacupuncture.tw. |
| 22 | electro-acupuncture.tw. |
| 23 | ear acupuncture.tw. |
| 24 | Auricular acupuncture.tw. |
| 25 | scalp acupuncture.tw. |
| 26 | plum blossom needle.tw. |
| 27 | fire needling.tw. |
| 28 | dermal needle.tw. |
| 29 | or/17-28 |
| 30 | Randomized controlled trial/ |
| 31 | randomization/ |
| 32 | Controlled study/ |
| 33 | Clinical trial/ |
| 34 | controlled clinical trial/ |
| 35 | Double Blind Procedure/ |
| 36 | Single Blind Procedure/ |
| 37 | Crossover Procedure/ |
| 38 | or/30-37 |
| 39 | (clinica$ adj3 trial$).mp. |
| 40 | ((singl$ or doubl$ or trebl$ or tripl$) adj3 (mask$ or blind$ or method$)).mp. |
| 41 | exp Placebo/ |
| 42 | placebo$.mp. |
| 43 | random$.mp. |
| 44 | ((control$ or prospectiv$) adj3 (trial$ or method$ or stud$)).mp. |
| 45 | (crossover$ or cross-over$).mp. |
| 46 | or/39-45 |
| 47 | 38 or 46 |
| 48 | exp ANIMAL |
| 49 | Nonhuman/ |
| 50 | Human/ |
| 51 | 48 or 49 |
| 52 | 51 not 50 |
| 53 | 47 not 52 |
| 54 | 16 and 29 and 53 |

Supplementary Table 2. Search strategy used in CENTRAL (via The Cochrane Library)

| **No.** | **Search items** |
| --- | --- |
| #1 | MeSH descriptor Asthma explode all trees |
| #2 | (asthma*) |
| #3 | (antiasthma* or anti-asthma*) |
| #4 | MeSH descriptor Respiratory Sounds, this term only |
| #5 | (wheez*) |
| #6 | MeSH descriptor Bronchial Spasm, this term only |
| #7 | (Bronchospas*) |
| #8 | (bronch* near/3 spasm*) |
| #9 | (Bronchoconstrict*) |
| #10 | MeSH descriptor Bronchoconstriction explode all trees |
| #11 | (bronch* near/3 constrict*) |
| #12 | MeSH descriptor Bronchial Hyperreactivity, this term only |
| #13 | MeSH descriptor Respiratory Hypersensitivity, this term only |
| #14 | (bronchial* or respiratory or airway* or lung*) near/3 (hypersensitive* or hyperreactive* or allerg* or insufficien*) |
| #15 | (dust or mite*) near/3 (allerg* or hypersensitive*) |
| #16 | #1 or #2 or #3 or #4 or #5 or #6 or #7 or #8 or #9 or #10 or #11 or #12 or #13 or #14 or #15 |
| #17 | MeSH descriptor Acupuncture explode all trees |
| #18 | Acupuncture:ti,ab,kw |
| #19 | Acupoints:ti,ab,kw |
| #20 | manual acupuncture:ti,ab,kw |
| #21 | Electroacupuncture:ti,ab,kw |
| #22 | electro-acupuncture:ti,ab,kw |
| #23 | ear acupuncture:ti,ab,kw |
| #24 | Auricular acupuncture:ti,ab,kw |
| #25 | scalp acupuncture:ti,ab,kw |
| #26 | plum blossom needle:ti,ab,kw |
| #27 | fire needling:ti,ab,kw |
| #28 | dermal needle:ti,ab,kw |
| #29 | #17 or #18 or #19 or #20 or #21 or #22 or #23 or #24 or #25 or #26 or #27 or #28 |
| #30 | #16 and #29 |

Supplementary Table 3. Search strategy used in SinoMed

| **No.** | **Search items** |
| --- | --- |
| #1 | "哮喘"[扩展:不加权] |
| #2 | 喘息 OR 喘证 OR 喘鸣 OR 哮鸣 OR 哮证 |
| #3 | (#2) OR (#1) |
| #4 | "针灸疗法"[扩展:不加权] |
| #5 | 针刺 OR 电针 OR 耳针 OR 头皮针 OR 梅花针 OR 火针 OR 皮肤针 |
| #6 | (#4) OR (#5) |
| #7 | "随机对照试验"[扩展:不加权] |
| #8 | 临床试验 OR 多中心研究 OR 临床观察 OR 随机 OR 试验 |
| #9 | (#7) OR (#8) |
| #10 | (#3) AND (#6) AND (#9) |

Supplementary Table 4. Search strategy used in China National Knowledge Infrastructure (CNKI), The Chinese Scientific Journal Database (VIP database), and the Wanfang database

| CNKI | (SU='哮喘' OR SU='喘息' OR SU='喘证' OR SU='喘鸣' OR SU='哮鸣' OR SU='哮病') AND (SU='针灸' OR SU='针刺' OR SU='电针' OR SU='耳针' OR SU='头皮针' OR SU='梅花针' OR SU='火针' OR SU='皮肤针') AND (SU='随机对照试验' OR SU='临床观察' OR SU='随机' OR SU='临床试验' OR SU='试验') |
| --- | --- |
| VIP database | (M=(哮喘+喘息+喘证+喘鸣+哮鸣+哮病)+R=(哮喘+喘息+喘证+喘鸣+哮鸣+哮病))*(M=(针灸+针刺+电针+耳针+头皮针+梅花针+火针+皮肤针)+R=(针灸+针刺+电针+耳针+头皮针+梅花针+火针+皮肤针))*(M=(随机对照试验+临床观察+随机+临床试验+试验)+R=(随机对照试验+临床观察+随机+临床试验+试验)) |
| Wanfang database | 主题:(哮喘 or 喘息 or 喘证 or 喘鸣 or 哮鸣 or 哮病)*主题:(针灸 or 针刺 or 电针 or 耳针 or 头皮针 or 梅花针 or 火针 or 皮肤针)*主题:(随机对照试验 or 临床观察 or 随机 or 临床试验 or 试验) |
